# Metformin use and the risk of incident immune-mediated diseases in patients with type 2 diabetes: a population-based cohort study

**DOI:** 10.3389/fimmu.2026.1768882

**Published:** 2026-03-24

**Authors:** Qianru Zhang, Fu-Shun Yen, Chuanhui Xu, Cheng-Li Lin, Hei-Tung Yip, Chii-Min Hwu, James Cheng-Chung Wei, Chih-Cheng Hsu

**Affiliations:** 1 Department of Rheumatology and Immunology, Beijing Tsinghua Changgung Hospital, School of Clinical Medicine, Tsinghua University, Beijing, China; 2 Dr. Yen’s Clinic, Taoyuan, Taiwan; 3 Department of Rheumatology, Allergy and Immunology, Tan Tock Seng Hospital, Singapore, Singapore; 4 Lee Kong Chian School of Medicine, Nanyang Technological University, Singapore, Singapore; 5 Management office for Health Data, China Medical University Hospital, Taichung, Taiwan; 6 College of Medicine, China Medical University, Taichung, Taiwan; 7 Section of Endocrinology and Metabolism, Department of Medicine, Taipei Veterans General Hospital, Taipei, Taiwan; 8 Faculty of Medicine, National Yang Ming Chiao Tung University School of Medicine, Taipei, Taiwan; 9 Department of Allergy, Immunology & Rheumatology, Chung Shan Medical University Hospital, Taichung, Taiwan; 10 Institute of Medicine, Chung Shan Medical University, Taichung, Taiwan; 11 Graduate Institute of Integrated Medicine, China Medical University, Taichung, Taiwan; 12 Institute of Population Health Sciences, National Health Research Institutes, Zhunan, Taiwan; 13 Department of Health Services Administration, China Medical University, Taichung, Taiwan; 14 Department of Family Medicine, Min-Sheng General Hospital, Taoyuan, Taiwan; 15 National Center for Geriatrics and Welfare Research, National Health Research Institutes, Huwei, Taiwan

**Keywords:** cohort study, immune-mediated diseases, incidence, metformin, type 2 diabetes mellitus

## Abstract

**Objectives:**

To investigate the association between metformin use and risks of incident immune-mediated diseases (IMDs) among patients with type-2 diabetes mellitus (T2DM).

**Methods:**

We conducted a retrospective cohort study using the National Health Insurance Research Database, including patients newly diagnosed with T2DM between 2000 and 2017 and followed until the end of 2018. Patients receiving metformin for ≥28 days were identified as users, and propensity score matching (1:1) was applied to balance baseline characteristics. The primary outcome was incident IMDs. Secondary outcomes included IMD-related hospitalization and all-cause mortality. Dose-response analyses were performed according to cumulative metformin exposure (<182 days, 182–364 days, >364 days). Hazard ratios (HRs) and 95% confidence intervals (CIs) were estimated using Cox proportional hazards models.

**Results:**

After propensity score matching, 20,460 metformin users and 20,460 non-users were included. Metformin use was associated with a higher risk of incident IMDs (adjusted HR 2.36, 95%CI 2.09-2.67). Metformin users also had a higher risk of IMD-related hospitalization (adjusted HR 2.44, 95%CI 1.94-3.05), but a lower observed risk of all-cause mortality (adjusted HR 0.64, 95%CI 0.60-0.68) compared with non-users. Longer cumulative metformin exposure was associated with progressively higher risks of IMDs and IMD-related hospitalization, whereas all-cause mortality decreased with longer use.

**Conclusion:**

In patients with T2DM, metformin use was associated with increased risks of incident IMDs and IMD-related hospitalization but a lower observed risk of all-cause mortality. These findings highlight the dual immunometabolic effects of metformin and underscore the need for individualized monitoring and further mechanistic research.

## Highlights

- Metformin use was associated with a 2.36-fold higher risk of developing immune-mediated diseases (IMD) in patients with type 2 diabetes mellitus (T2DM) than metformin non-users.
- Metformin users had a 2.44-fold higher risk of IMD-related hospitalization but a 36% lower risk of all-cause mortality.
- Longer metformin exposure was associated with progressively higher risks of IMDs and IMD-related hospitalization, but lower risk of all-cause mortality.

## 1 Introduction

Type 2 diabetes mellitus (T2DM) is a chronic metabolic disorder characterized by insulin resistance and persistent low-grade inflammation (1, 2). This metabolic-immune imbalance may increase susceptibility to immune-mediated diseases (IMDs) (3, 4). Metformin, the most widely prescribed glucose-lowering agent for T2DM, exerts immunomodulatory and anti-inflammatory effects through activation of AMP-activated protein kinase and inhibition of the mTOR pathway (5, 6). However, evidence regarding metformin’s effects on autoimmunity remains inconsistent, with some studies suggesting a protective role (7, 8) while others reporting no significant association (9, 10). Despite its wide use and biological plausibility, population-based evidence on the relationship between metformin use and new-onset IMDs among T2DM patients are limited.

Clarifying the association has important clinical and public health implications. Metformin is the first-line therapy for T2DM and exerts immunometabolic effects beyond glucose regulation, including modulation of inflammation and oxidative stress (11). Understanding whether these immunometabolic effects translate into altered risks of IMDs could refine individualized diabetes management and inform drug repurposing strategies (12, 13). Moreover, elucidating metformin’s role in immune regulation may provide new insights into the shared pathways linking metabolic dysfunction and autoimmunity, with potential implications for both disease prevention and therapeutic innovation.

Therefore, this study aimed to investigate the association between metformin use and the risk of developing new-onset IMDs among patients with T2DM. We further assessed subsequent hospitalization and all-cause mortality risk. We hypothesized that metformin users among T2DM patients would have a decreased risk of incident IMDs than metformin non-users. By leveraging a large population-based cohort and applying rigorous control for confounding, this study seeks to provide comprehensive evidence on the long-term immunologic effects of metformin in patients with T2DM.

## 2 Materials and methods

### 2.1 Study design and participants

This retrospective cohort study was conducted using data from Taiwan’s National Health Insurance Research Database (NHIRD), which contained information on beneficiaries’ residential area, age, sex, premium, diagnoses, prescriptions, and medical procedures for more than 20 million people (14). Diagnoses were recorded according to the *International Classification of Diseases, Ninth and Tenth Revision, Clinical Modification* (ICD-9-CM and ICD-10-CM). The NHIRD is linked to the National Death Registry to ascertain mortality information. The study protocol was approved by the Research Ethics Committee of China Medical University and Hospital (CMUH109-109-REC2-031). All identifiable information of healthcare providers and patients was encrypted before release to ensure privacy protection, therefore, the requirement for informed consent was waived by the Research Ethics Committee.

### 2.2 Identification of T2DM

Patients with T2DM were identified using a validated algorithm based on ICD codes (ICD-9 code: 250, except 250.1x; ICD-10 code: E11), requiring at least three outpatient visits or one hospitalization record of T2DM within a one-year observational window, occurring at any time during the study identification period from January 1, 2000, to December 31, 2017, which achieved 74.6% accuracy (15).

Patients were excluded if they were younger than 20 or older than 80 years, or if they had a prior diagnosis of IMDs before the index date.

### 2.3 Exposure

Among patients with T2DM, those who had a cumulative metformin prescription duration of ≥28 days, calculated as the sum of all metformin prescription days (including intermittent or non-continuous use), were classified as the exposed group. Patients who did not receive metformin during the follow-up period served as the unexposed group. To ensure a clear distinction between exposure groups, patients who received metformin for less than 28 days were excluded.

Metformin exposure was treated as a time-fixed variable, defined at cohort entry based on cumulative prescription duration. Cumulative metformin exposure was assessed from the index date until the occurrence of IMD, IMD-related hospitalization, death, or the end of follow-up (December 31, 2018), whichever came first. Metformin prescriptions issued after an IMD event were not included.

The index date for the exposed group was defined as the date of the first metformin prescription. For the unexposed group, a corresponding index date was assigned by matching the same interval from T2DM diagnosis to the index date of the paired exposed patient.

### 2.4 Outcomes

The primary outcome was the first occurrence of IMDs. Secondary outcomes included IMD-related hospitalization and all-cause mortality. IMDs and IMD-related hospitalization were identified using ICD-9 and ICD-10 codes (**Supplementary Table 1**). Mortality was ascertained either by in-hospital death documented at the time of discharge, with the discharge date recorded as the date of death, or by termination of National Health Insurance coverage following hospital discharge due to a catastrophic illness, in the absence of any subsequent healthcare utilization for more than one year. In the latter scenario, the date of insurance termination was considered the date of death. For each outcome, follow-up time was calculated separately from the index date to the occurrence of the corresponding event, death, or the end of follow-up on December 31, 2018, whichever came first.

### 2.5 Covariates

This study included the following covariates, assessed 365 days prior to the index date based on ICD codes (**Supplementary Table 2**): age, sex, body mass index (BMI) category, smoking, alcohol-related disorders, and comorbidities including hypertension (HT), dyslipidemia, coronary artery disease (CAD), stroke, atrial fibrillation, peripheral arterial occlusive disease (PAOD), chronic kidney disease (CKD), retinopathy, chronic obstructive pulmonary disease (COPD), gout, hepatitis, liver cirrhosis, cancer, psychosis, depression, and dementia. T2DM duration was defined as the time interval between the first recorded diagnosis of T2DM and the index date. We also calculated the Charlson Comorbidity Index (CCI) and Diabetes Complication Severity Index (DCSI) score (13, 14) to quantify overall comorbidity burden and T2DM severity. Medication use was further included, encompassing sulfonamides, thiazolidinedione (TZD), dipeptidyl peptidase-4 inhibitors (DPP-4i), alpha-glucosidase inhibitor (AGI), glucagon-like peptide-1 receptor agonists (GLP-1 RAs), number of oral antidiabetic drugs (OAD), insulin, corticosteroids, immunosuppressants, statin, non-steroid anti-inflammatory drugs (NSAIDs), and aspirin.

### 2.6 Statistical analysis

To ensure comparability of baseline characteristics between cohorts, we applied a non-parsimonious multivariable logistic regression model to generate propensity scores and conducted 1:1 matching (PSM) using greedy nearest neighbor matching based on age, sex, comorbidities, medications, and T2DM duration. Matching quality was considered acceptable when no statistically significant differences (p > 0.05) were observed between groups after matching.

Incidence rates per 1,000 person-years were calculated for each outcome. To account for potential differences in follow-up duration between groups due to outcome occurrence, person-years were used in the calculation of incidence rates, and Cox proportional hazards models were applied to appropriately handle varying follow-up times. Crude and multivariable-adjusted Cox proportional hazards models were then applied to the propensity score-matched cohorts to estimate the hazard ratios (HRs) and 95% confidence intervals (CIs). Analyses based on cumulative duration of metformin use (<182, 182-364, and >364 days) were conducted to investigate a potential duration-response relationship with the risks of IMDs, IMD-related hospitalization, and death.

All statistical analyses were conducted using SAS (version 9.4; SAS Institute, Cary, NC, USA), and two-tailed p-values <0.05 were considered statistically significant.

## 3 Results

### 3.1 Baseline characteristics

**Figure 1:**
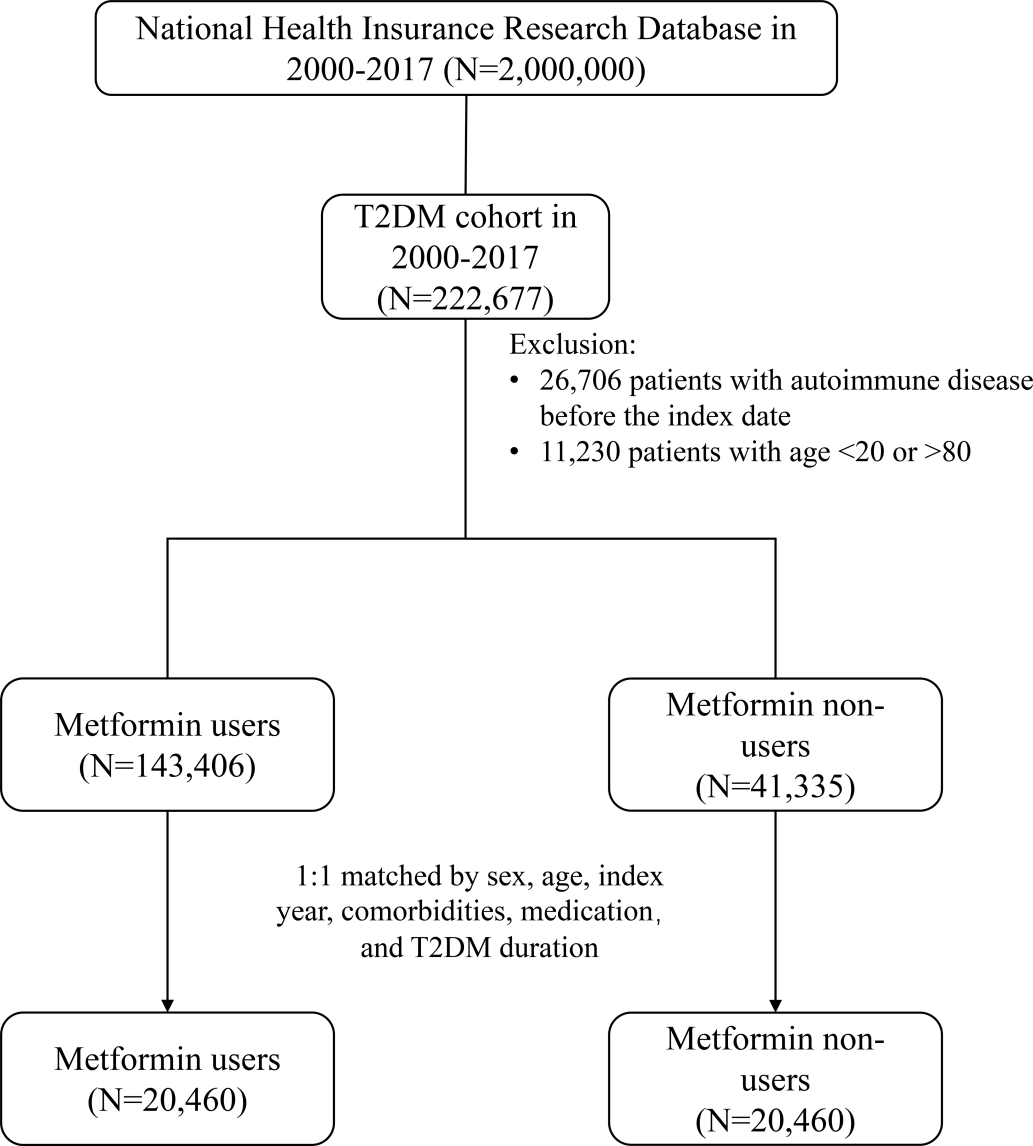
Flow diagram of the study population selection from the type 2 diabetes mellitus (T2DM) cohort. This flow diagram illustrates the selection process for metformin users and non-users from the National Health Insurance Research Database. Individuals with type 2 diabetes mellitus (T2DM) were screened, and those with a prior immune-mediated diseases, younger than 20 years, or older than 80 years were excluded. After 1:1 propensity score matching, a total of 20,460 metformin users and 20,460 metformin non-users were included in the final analytic cohort. T2DM indicates type 2 diabetes mellitus.

After propensity score matching, 20,460 patients with T2DM were identified in both metformin user and non-user cohorts (**Figure 1**). Baseline characteristics of the matched cohorts are shown in **Table 1**. The mean age was approximately 59 years, and about half of the patients were female. Both cohorts were well-balanced regarding the age, sex, BMI category, smoking, alcohol-related disorders, comorbidities, and most medications. CCI, DCSI, and the number of OAD were also similarly distributed. The mean follow-up duration for incident IMDs was comparable between metformin users and nonusers (4.41 vs. 4.37 years, p=0.29), whereas follow-up periods were slightly longer among metformin users for hospitalization (4.66 vs. 4.43 years, p<0.001) and death (4.73 vs. 4.44 years, p<0.001).

**Table 1:** Baseline characteristics of patients with T2DM by metformin use.

Variables	Metformin non-user n=20,460	Metformin user n=20,460	p-value
Age, n (%)			0.51
20–40 years	1,590 (7.8)	1652 (8.1)	
41–60 years	9277 (45.3)	9224 (45.1)	
61–80 years	9593 (46.9)	9584 (46.8)	
Age, mean (SD), years	58.84 (12.05)	58.79 (12.06)	0.71
Sex, n (%)			0.43
Female	10346 (50.6)	10426 (51.0)	
Male	10114 (49.4)	10034 (49.0)	
Body mass index category, n (%)			
Overweight	534 (2.6)	532 (2.6)	0.95
Obesity	428 (2.1)	434 (2.1)	0.84
Severe obesity	76 (0.4)	70 (0.3)	0.62
Smoking, n (%)	583 (2.8)	562 (2.7)	0.53
Alcohol disorders, n (%)	1192 (5.8)	1178 (5.8)	0.77
Comorbidities, n (%)			
Hypertension	13070 (63.9)	13073 (63.9)	0.98
Dyslipidemia	13255 (64.8)	13257 (64.8)	0.98
Coronary artery disease	6249 (30.5)	6267 (30.6)	0.85
Stroke	3207 (15.7)	3133 (15.3)	0.31
Atrial fibrillation	21 (0.1)	21 (0.1)	1.00
PAOD	831 (4.1)	836 (4.1)	0.90
CKD	2701 (13.2)	2639 (12.9)	0.36
Retinopathy	1587 (7.8)	1549 (7.6)	0.48
COPD	5861 (28.6)	5864 (28.7)	0.97
Gout	5528 (27)	5508 (26.9)	0.82
Hepatitis	1910 (9.3)	1904 (9.3)	0.92
Liver cirrhosis	464 (2.3)	466 (2.3)	0.95
Cancer	929 (4.5)	921 (4.5)	0.85
Psychosis	404 (2.0)	423 (2.1)	0.50
Depression	7376 (36.1)	7383 (36.1)	0.94
Dementia	666 (3.3)	643 (3.1)	0.52
CCI, n (%)			0.27
1	4190 (20.5)	4128 (20.2)	
2-3	10187 (49.8)	10350 (50.6)	
	6083 (29.7)	5982 (29.2)	
DCSI, n (%)			0.91
0	6965 (34.0)	6926 (33.9)	
1	3690 (18.0)	3712 (18.1)	
≥2	9805 (47.9)	9822 (48.0)	
Medication, n (%)			
Sulfonamides	2042 (10.0)	826 (4.0)	<0.0001
Thiazolidinedione	361 (1.8)	140 (0.7)	<0.0001
DPP-4 inhibitors	878 (4.3)	345 (1.7)	<0.0001
AGI	849 (4.1)	163 (0.8)	<0.0001
GLP-1 RAs	14 (0.1)	15 (0.1)	0.85
Number of OAD			0.93
0-1	19468 (95.2)	19468 (95.2)	
2-3	960 (4.7)	957 (4.7)	
>3	32 (0.2)	35 (0.2)	
Insulin	4164 (20.4)	4174 (20.4)	0.90
Corticosteroid	79 (0.4)	65 (0.3)	0.24
Immunosuppressants	54 (0.3)	59 (0.3)	0.64
Statin	10186 (49.8)	10135 (49.5)	0.61
NSAIDs	17159 (83.9)	17269 (84.4)	0.14
Aspirin	6357 (31.1)	6273 (30.7)	0.37
T2DM duration, mean (SD), years	3.16 (3.62)	2.87 (3.90)	<0.0001
Follow up time, mean (SD), years			
IMD	4.37 (3.82)	4.41 (3.75)	0.29
IMD-related hospitalization	4.43 (3.86)	4.66 (3.90)	<0.0001
All-cause mortality	4.44 (3.87)	4.73 (3.95)	<0.0001

AGI, alpha-glucosidase inhibitor; BMI, body mass index; CAD, coronary artery disease; CCI, Charlson comorbidity index; CKD, chronic kidney disease; COPD, chronic obstructive pulmonary disease; DCSI, diabetes complications severity index; DPP-4 inhibitors, dipeptidyl peptidase-4 inhibitors; GLP-1 RAs, glucagon-like peptide-1 receptor agonists; IMD, immune-mediated disease; NSAIDs, non-steroidal anti-inflammatory drugs; OAD, oral antidiabetic drug; PAOD, peripheral arterial occlusive disease; SD, standard deviation; SGLT2 inhibitors, sodium-glucose cotransporter-2 inhibitors; T2DM, type 2 diabetes mellitus.

Note: Follow-up time was defined as the interval from the index date to the first occurrence of the outcome of interest (IMD or IMD-related hospitalization), death, or the end of the observation period (December 31, 2018), whichever occurred first.

### 3.2 Main outcomes

After propensity score matching, the metformin user cohort had a significantly higher risk of developing immune-mediated diseases (**Table 2**; 10.45 vs. 4.08 per 1,000 person-years, adjusted HR 2.36, 95%CI 2.09-2.67) and IMDs-related hospitalization (3.12 vs. 1.14 per 1,000 person-years, adjusted HR 2.44, 95%CI 1.94-3.05), but a lower risk of all-cause mortality (19.38 vs. 27.61 per 1,000 person-years, adjusted HR 0.64, 95%CI 0.60-0.68) compared with the metformin non-user cohort among patients with T2DM.

**Table 2:** Incidence rates and hazard ratios of immune-mediated diseases in patients with T2DM by metformin use.

	Metformin non-user		Metformin user			
Outcomes	n	Incidence rate/1,000 person-years	n	Incidence rate/1,000 person-years	Crude HR (95% CI)	Adjusted* HR (95% CI)
IMDs	365	4.08	943	10.45	2.56 (2.26, 2.88)	**2.36 (2.09, 2.67)**
IMD related hospitalization	103	1.14	297	3.12	2.75 (2.20, 3.44)	**2.44 (1.94, 3.05)**
All-cause mortality	2510	27.61	1876	19.38	0.70 (0.66, 0.74)	**0.64 (0.60, 0.68)**

IMDs, immune-mediated diseases; HR, hazard ratio; CI, confidence interval.

*Adjusted HR: Model adjusted for age groups, sex, comorbidities, medications, and duration of type 2 diabetes as listed in **Table 1**.

Note: For each outcome, person-years were calculated from the index date to the occurrence of the corresponding event, death, or the end of follow-up (December 31, 2018), whichever came first.

Bold values indicate statistically significant results.

### 3.3 Dose-response analysis

According to cumulative duration of metformin use, compared with non-users, patients with longer metformin exposure had higher adjusted risks of IMDs (**Table 3**): adjusted HRs were 1.34 (95%CI 1.12-1.60) for <182 days, 1.46 (95%CI 1.12-1.91) for 182–364 days, and 3.17 (95%CI 2.79-3.61) for >364 days. Similarly, the risk of IMD-related hospitalization increased with cumulative metformin duration: adjusted HRs were 1.82 (95%CI 1.34-2.48), 2.13 (95%CI 1.38-3.29), and 2.82 (95%CI 2.22-3.58) for the respective exposure categories. In contrast, all-cause mortality decreased with longer metformin use compared with non-users, adjusted HRs were 0.93 (95%CI 0.86-1.01) for <182 days, 0.85 (95%CI 0.74-0.98) for 182–364 days, and 0.47 (95%CI 0.44-0.51) for >364 days.

**Table 3:** Incidence rates and hazard ratios of immune-mediated diseases in patients with T2DM by cumulative duration of metformin use.

IMDs					
Variables	n	Incidence rate / 1,000 person-years	Crude HR (95% CI)		Adjusted* HR (95% CI)
Metformin non-user_drug days	365	4.08	1.00 (Ref)		1.00 (Ref)
Metformin user_drug days					
<182	180	6.81	1.68 (1.41, 2.01)		**1.34 (1.12, 1.60)**
182-364	64	7.97	1.81 (1.39, 2.36)		**1.46 (1.12, 1.91)**
>364	699	12.53	3.08 (2.72, 3.50)		**3.17 (2.79, 3.61)**

IMD-related hospitalization					
Variables	n	Incidence rate / 1,000 person-years	Crude* HR (95% CI)		Adjusted** HR (95% CI)
Metformin non-user_drug days	103	1.14	1.00 (Ref)		1.00 (Ref)
Metformin user_drug days					
<182	70	2.58	2.25 (1.66, 3.05)		**1.82 (1.34, 2.48)**
182-364	26	3.14	2.64 (1.72, 4.06)		**2.13 (1.38, 3.29)**
>364	201	3.36	3.00 (2.36, 3.80)		**2.82 (2.22, 3.58)**

All-cause mortality					
Variables	n	Incidence rate / 1,000 person-years	Crude* HR (95% CI)	Adjusted** HR (95% CI)	
Metformin non-user_drug days	2,510	27.61	1.00 (Ref)	1.00 (Ref)	
Metformin user_drug days					
<182	802	29.27	1.04 (0.96, 1.13)	0.93 (0.86, 1.01)	
182-364	221	26.43	0.96 (0.83, 1.10)	**0.85 (0.74, 0.98)**	
>364	853	13.97	0.51 (0.47, 0.55)	**0.47 (0.44, 0.51)**	

IMDs, immune-mediated diseases; HR, hazard ratio; CI, confidence interval.

*Adjusted HR: Model adjusted for age groups, sex, comorbidities, medications, and duration of type 2 diabetes as listed in **Table 1**.

Bold values indicate statistically significant results.

## 4 Discussion

In this large population-based cohort study of patients with T2DM, metformin use was associated with a higher risk of incident IMDs and IMD-related hospitalization, but with a lower observed risk of all-cause mortality. Dose-response analyses further demonstrated prolonged cumulative metformin use was associated with progressively higher risks of IMDs and related hospitalization, while it was simultaneously linked to a lower observed risk of mortality. These findings indicate that metformin is associated with a higher risk of immune-mediated events while being linked to improved survival outcomes in patients with T2DM.

Our study found metformin use was associated with a higher risk of developing IMDs among patients with T2DM, including first onset and hospitalization. This finding was contrary to our initial hypothesis that metformin would reduce the risk of autoimmune diseases. Although preclinical studies have generally highlighted immunosuppressive or immunoregulatory effects of metformin (16–19), our findings suggest a more complex scenario in the context of T2DM due to insulin resistance, chronic low-grade inflammation, and immune-metabolic imbalance (20, 21). In this vulnerable setting, chronic metformin exposure may perturb immune homeostasis via multiple interconnected mechanisms: modifying gut microbiota composition, including expansion of short-chain fatty acids (SCFA)-producing bacteria such as *Akkermansia muciniphila (*22, 23), and altering intestinal barrier integrity, thereby influencing systemic antigen exposure (24); affecting innate immune cells by shifting macrophage polarization and cytokine profiles (25), and reprogramming dendritic cells toward tolerogenic or immunostimulatory states (26); and modulating adaptive immunity by altering T-cell subset balance, including Th17/Treg ratios, enhancing CD8+ T-cell activation (27), and potentially influencing B-cell metabolism and autoantibody production. Collectively, these mechanisms may undermine immune tolerance and predispose T2DM patients to autoimmune disorders under chronic metabolic stress.

Conversely, metformin use was associated with a lower risk of all-cause mortality among patients with T2DM. This survival benefit is consistent with extensive epidemiological and mechanistic evidence showing that metformin improves metabolic health and mitigates age-related and cardiovascular mortality risks (28, 29). Beyond glycemic control, metformin enhances endothelial function, reduces oxidative stress, and attenuates systemic inflammation through both AMP-activated protein kinase (AMPK)-dependent and independent signaling (30, 31). In addition, metformin enhances mitochondrial function and autophagy, attenuates cellular senescence, and modulates lipid metabolism, thereby reducing cardiovascular and cancer-related mortality (32–34). Therefore, while long-term metformin exposure might disturb immune tolerance and increase autoimmune susceptibility in metabolically stressed individuals, its pleiotropic metabolic and vascular benefits appear to outweigh these risks, resulting in improved overall survival among individuals with T2DM. These dual and context-dependent effects underscore the complex immunometabolic actions of metformin and highlight the need to balance its metabolic benefits against potential immune-related risks in long-term use.

Dose-response analyses provided further insight into this dual relationship. The observation that longer cumulative metformin use corresponded to higher IMD risk, yet lower mortality suggests that the associations may vary with treatment duration and clinical context. While one possible explanation is that chronic treatment may amplify immune disturbances driven by metabolic stress and immunosenescence, alternative explanations should also be considered. In particular, patients receiving long-term metformin are likely to have more frequent healthcare encounters, which may increase opportunities for IMD detection and contribute to surveillance bias. In addition, protopathic bias cannot be fully excluded, as early or subclinical immune manifestations may precede formal diagnosis and influence treatment persistence. In contrast, sustained metabolic stabilization likely dominates long-term survival outcomes (35, 36). These findings highlight the heterogeneous effects of metformin across different outcomes and underscore the need for cautious interpretation of dose–response patterns, particularly in observational settings.

Our study has several strengths. First, it utilized large, nationwide, population-based cohorts with extended follow-up which could provide sufficient statistical power to real world clinical practice. The use of comprehensive administrative and prescription databases enabled accurate identification of metformin exposure, comorbidities, and incident IMDs. In addition, the linkage to the National Death Registry ensured complete and accurate capture of mortality data, allowing robust assessment of survival outcomes. Second, the application of propensity score matching effectively balanced baseline characteristics between metformin users and non-users, thereby minimizing confounding by indication. Third, the analysis of cumulative metformin exposure also provided insights into potential dose-response relationships between cumulative metformin use and the risks of IMDs, IMD-related hospitalization, and mortality.

Nevertheless, several limitations should be acknowledged. First, despite rigorous matching, residual confounding from unmeasured variables could not be fully excluded. In particular, key indicators of disease severity and metabolic control, such as HbA1c levels, fasting glucose, renal function parameters, and body mass index, were not available in the database. These factors may influence both metformin prescribing patterns and the risk of immune-mediated diseases. However, to partially address this limitation, we incorporated the DCSI, the number of concomitant oral antidiabetic agents, and insulin use as proxy measures of disease severity and glycemic control in the propensity score matching process. This approach was intended to minimize imbalance in glycemic control between the metformin and comparison groups. Moreover, information on metformin adherence, dosage adjustments, variations in initial therapy choice, and concurrent lifestyle factors such as diet and physical activity were not available. In particular, data on sodium-glucose cotransporter-2 inhibitors (SGLT2is) were limited, as these agents were introduced in Taiwan in 2016, and our follow-up ended in 2018. The small number of SGLT2i users, especially after matching, prevented adequate control for their potential effects. Additionally, although propensity score matching effectively balanced most baseline characteristics between groups, some clinically relevant variables, particularly T2DM duration and the use of specific glucose-lowering agents, remained imbalanced after matching. These variables may partially reflect differences in diabetes severity, disease progression, or treatment intensity, which could contribute to residual confounding. To mitigate potential residual confounding from these discrepancies, we included these variables in the multivariable-adjusted Cox models for further adjustment. Nonetheless, imperfect matching in these factors may still have introduced minor bias, and the results should therefore be interpreted with caution. Importantly, because propensity score matching resulted in the inclusion of only a subset of eligible patients with T2DM (40,920 of 222,677; approximately 18%), the generalizability of our findings may be limited. The matched cohort may represent a more selected population with greater overlap in baseline characteristics, and the observed associations may not be fully applicable to patients excluded from matching. Furthermore, detailed comparisons between included and excluded individuals could not be presented, which may further constrain interpretability. Second, if non-users were treatment-naïve (i.e., oral antidiabetic drug use = 0), these individuals may have adhered to more intensive lifestyle interventions, such as stricter dietary control and physical activity, which could potentially contribute to a lower risk of immune-mediated diseases. Third, the identification of IMDs and related hospitalizations relied on administrative claims and ICD coding, which may introduce misclassification bias, although these codes have been validated in previous research. Furthermore, we recognize that the aggregation of IMDs may mask heterogeneity across individual disease categories. Therefore, our results should be interpreted as indicating an association with the overall risk of IMDs rather than with any specific immune-mediated condition. Fourth, the retrospective nature of the study and reliance on data from a single healthcare system may limit generalizability to other settings or other populations. Finally, the cohort was restricted to patients with T2DM, and the findings may not be generalizable to non-diabetic populations or those with type 1 diabetes.

In conclusion, metformin use among patients with T2DM was associated with a higher risk of incident IMDs and related hospitalization but a lower risk of all-cause mortality. Further, the observed dose-response pattern with cumulative metformin use may reflect the complex and context-dependent immunometabolic associations of the drug. These results emphasize the need for further research to clarify the mechanisms underlying the observed dual associations with autoimmunity and survival, which may help inform future personalized management strategies and support exploration of potential therapeutic applications in T2DM in observational settings.

## Data Availability

The original contributions presented in the study are included in the article/**Supplementary Material**, further inquiries can be directed to the corresponding author/s.
