# Supplementary material for: Metformin use and the risk of incident immune-mediated diseases in patients with type 2 diabetes: a population-based cohort study
Source: Front Immunol. 2026 Mar 24;17:1768882. doi: 10.3389/fimmu.2026.1768882 (PMC13055625; doi:10.3389/fimmu.2026.1768882)
Supplement: Supplementary file 1 [file Table1.docx]

| **Supplementary Table 1.** The International Classification of Diseases, Ninth and Tenth Revision, Clinical Modification (ICD-9-CM and ICD-10-CM) codes of immune-mediated diseases used in this study | | |
| --- | --- | --- |
| Conditions | ICD-9 | ICD-10 |
| Alopecia areata | 704.1 | L68 |
| Ankylosing spondylitis | 720 | M45.9 |
| Autoimmune hemolytic anemia | 283 | D59.0, D59.1 |
| Autoimmune disease, not elsewhere classified | 279.49 | D89.89 |
| Autoimmune hepatitis | 571.42 | K75.4 |
| Behcet’s disease | 136.1 | M35.2 |
| Biliary cirrhosis | 571.6 | K74.3, K74.4, K74.5 |
| Celiac disease | 579 | K90.0 |
| Cholangitis | 576.1 | K83.0 |
| Dermatomyositis | 710.3 | M33.9 |
| Graves’ disease | 242,242.01 | E05 |
| Guillain-Barre´syndrome | 357 | G61.0 |
| Hashimoto’s thyroiditis | 245.2 | E06.3 |
| Hereditary hemolytic anemia | 281 | D51.0 |
| Inflammatory bowel diseases | 555, 556 | K50, K51 |
| Juvenile rheumatoid arthritis | 714.30, 714.33 | M08 |
| Multiple sclerosis | 340 | G35 |
| Myasthenia gravis | 358 | G70.00, G70.01 |
| Pemphigus | 694.4 | L10 |
| Polyarteritis nodosa | 446 | D58.0 |
| Polymyalgia rheumatica | 725 | M35.3 |
| Polymyositis | 710.4 | M33.20, M33.29 |
| Psoriatic disease | 696.0, 696.1, 696.8 | L40 |
| Raynaud's syndrome | 443 | I73.00, I73.01 |
| Rheumatoid arthritis | 714 | M06.9 |
| Sarcoidosis | 135 | D86.9 |
| Systemic sclerosis | 710.1 | M34 |
| Sjogren's syndrome | 710.2 | M35 |
| Systemic lupus erythematosus | 710 | M32.10 |
| Uveitis | 364.00, 364.01 | H20.00, H20.011, H20.12, H20.13, H20.19 |
| Vasculitis | 443.1, 446.0, 446.1, 446.2, 446.4, 446.5, 446.7, | I73.1, M30, M31 |

| **Supplementary Table 2**. The International Classification of Diseases, Ninth and Tenth Revision, Clinical Modification (ICD-9-CM and ICD-10-CM) codes of covariates used in this study | | |
| --- | --- | --- |
| Conditions | ICD-9 | ICD-10 |
| Overweight | 278.02, 783.1, V85.2 | R63.5 |
| Obesity | 278.00, 649.1, V77.8, V85.3 | E66.09, E66.1, E66.8, E66.9, Z13.89 |
| Severe obesity | 278.01, 649.2, V45.86, V85.4 | E66.01, E66.2 |
| Smoking | 305.1, 649.0, V15.82 | F17.200, F17.201, F17.210, F17.220, F17.221, F17.290, F17.291, Z87.891 |
| Alcohol disorders | 291, 303, 305.0, 571.0-571.3, V11.3, V79.1 | F10, K70.40, K70.41, K70.9 |
| Hypertension | 401–405 and A26 | I10, I11, I12, I13, I15, N26.2 |
| Dyslipidemia | 272 | E71.30, E71.31, E71.32, E71.39, E75.21, E75.22, E75.23, E75.24, E75.25, E75.29, E75.3, E75.4, E75.5, E75.6, E77, E78.0, E78.1, E78.2, E78.3, E78.4, E78.5, E78.6, E78.70, E78.71, E78.72, E78.79, E78.8, E78.9 |
| Coronary artery disease | 410-414 | I20, I21, I22, I24, I25.1, I25.2, I25.3, I25.4, I25.5, I25.6, I25.7, I25.81, I25.82, I25.83, I25.84, I25.89, I25.9 |
| Stroke | 430-438 | G45.0, G45.1, G45.2, G45.3, G45.4, G45.8, G45.9, G46, I60, I61, I62, I63, I65, I66, I67.0, I67.1, I67.2, I67.3, I67.4, I67.5, I67.6, I67.7, I67.8, I67.9, I68, I69 |
| Atrial fibrillation | 427 | I45.0, I45.1, I45.2, I45.3, I45.4, I45.5, I45.6 |
| Peripheral arterial occlusive disease | 440.0, 440.20, 440.21, 440.22, 440.23, 440.24, 440.3, 440.4, 443.9, 443.81, 443.89 | I70.2, I70.92, I75.0, I73.9 |
| Chronic kidney disease | 250.4x, 403.xx, 404.xx, 585.xx, 586.xx, 581.8x,791.0x, 593.9x | E10.2, E10.65, E11.2, E11.65, E13.2, I12, I13, N03, N08, E10.21, E11.21, N05, N06, N07, N14, N15.0, N15.8, N15.9, N16, N17.1, N17.2, N18, N19 |
| Retinopathy | 362.02, 362.07, 362.0 | H35.0, E08.311-E08.359, E09.311-E09.359, E11.311-E11.359, E13.311-E13.359 |
| Chronic obstructive pulmonary disease | 491, 492, 496 | J41, J42, J44, J43, J44.9 |
| Gout | 274, V77.5 | M10, M1A |
| Hepatitis | ICD-9: 277.4, 570, 572.8, 573.3, 573.8, 576.8, 782.4 | B15.0-B19.9 |
| Liver cirrhosis | 571.5, 571.2, 571.6 | K70.2, K70.30, K70.31, K74.0, K74.1, K74.2, K74.60, K74.69, K74.3, K74.4, K74.5 |
| Cancer | 140-239 | C00-D49, except D00-D36, D3A |
| Psychosis | 290-299 | F20-29 |
| Depression | 311 | F32, F33 |
| Dementia | 290,290.4, 291.2, 292.82 and 331 | F03.90, F05, F02.80, F02.81, F01.50, F01.51, G30 |
